# Repurposing the Insecticide Flufenerim, the Only Anti-*Cryptococcus* Compound Identified in the MMV Global Health Priority Box, as a Candidate for the Environmental Control of the Cryptococcosis Agents

**DOI:** 10.1021/acsomega.6c04207

**Published:** 2026-10-06

**Authors:** Juliana Godoy, Iara Bastos de Andrade, Edinaldo Castro de Oliveira, Igor Avellar-Moura, Kamilla Xavier Gomes, Felipe Carvalho da Costa, Marcelo Kurgonas de Oliveira, Carlos Pelleschi Taborda, Allan Jefferson Guimarães, Rosely Maria Zancopé-Oliveira, Marcos Abreu Almeida, Susana Frases, Rodrigo Almeida-Paes

**Affiliations:** † 37903 Fundação Oswaldo Cruz, Instituto Nacional de Infectologia Evandro Chagas, Laboratório de Micologia, Rio de Janeiro, Rio de Janeiro 21040-900, Brazil; ‡ 28105 Universidade Federal de São Paulo, Departamento de Medicina, Divisão de Doenças Infecciosas, Laboratório Especial de Micologia, São Paulo 04021-001, Brazil; § 28125 Universidade Federal do Rio de Janeiro, Instituto de Biofísica Carlos Chagas Filho, Laboratório de Biofísica de Fungos, Rio de Janeiro, Rio de Janeiro 21941-853, Brazil; ∥ 28110 Universidade Federal Fluminense, Instituto Biomédico, Laboratório de Bioquímica e Imunologia das Micoses, Niterói, Rio de Janeiro 24210-130, Brazil; ⊥ 28133 Universidade de São Paulo, Instituto de Ciências Biológicas, Departamento de Microbiologia, Laboratório de Fungos Patogênicos Dimórficos, São Paulo, São Paulo 05508-900, Brazil; # Rede Micologia, Fundação de Amparo á Pesquisa do Estado do Rio de Janeiro (FAPERJ), Rio de Janeiro, Rio de Janeiro 20020-000, Brazil

## Abstract

*Cryptococcus neoformans* and *Cryptococcus gattii* are environmental fungi that cause cryptococcosis, a severe, life-threatening infection, particularly in immunocompromised individuals. Current treatments are limited by toxicity, restricted availability, and the emergence of resistance, highlighting the need for novel antifungal agents. Here, 240 compounds from the Medicines for Malaria Venture (MMV) Global Health Priority Box were screened for activity against these two species. Flufenerim, originally developed as an insecticide, was the only compound that exhibited inhibitory activity at 1 μM (0.364 μg/mL). Minimum inhibitory concentrations ranged from 0.03 μg/mL to >8 μg/mL across 15 isolates, and the compound exhibited a predominantly fungistatic profile, except for one *C. gattii* strain. At subinhibitory concentrations, flufenerim significantly reduced capsule thickness in both species, melanization in *C. neoformans*, and urease production in *C. gattii*, without affecting phospholipase activity. *In silico* ADMET predictions indicated predicted toxicological risks requiring further evaluation. Cytotoxicity assays further demonstrated that the compound did not exhibit significant toxicity in murine and human macrophages, nor in human brain endothelial cells (HBECs). Exposure of *Galleria mellonella* larvae to flufenerim produced sublethal physiological effects without mortality. Due to the unfavorable ADMET profile, we evaluated whether this compound could be used for environmental control of fungal reservoirs. Tree-hollow samples spiked with *C. neoformans* and treated with flufenerim yielded fewer yeast colonies than untreated controls when plated on Staib’s agar. These findings identify flufenerim as a new anti-*Cryptococcus* compound and provide preliminary evidence supporting its potential for reducing *C. neoformans* recovery from environmental substrates. However, the ecological complexity of *Cryptococcus* reservoirs and the potential effects on nontarget microorganisms require extensive investigation before any environmental application can be considered.

## Graphical abstract

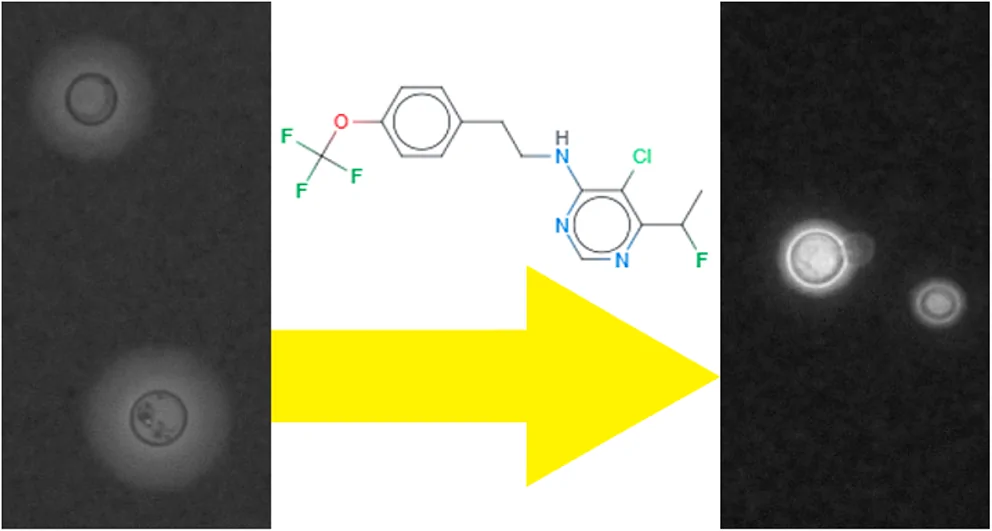

## 1 Introduction

Fungal infections have become an increasing global health concern, largely driven by climate change, environmental disturbances, and the rising number of immunocompromised individuals.^1,2^ Among systemic fungal diseases, cryptococcosis stands out for its clinical severity and high mortality rates, particularly in low-income regions with limited access to diagnosis and treatment.^3^ Caused primarily by *Cryptococcus neoformans* and *Cryptococcus gattii*, this opportunistic mycosis affects both immunocompetent and immunocompromised individuals, including people living with HIV/AIDS, cancer patients, transplant recipients, and those undergoing long-term immunosuppressive therapy.^4,5^

*Cryptococcus* spp. are encapsulated yeasts with a complex arsenal of virulence factors, such as capsule polysaccharides, melanin production, secretion of hydrolytic enzymes (such as phospholipases, proteases, urease), reactive oxygen species detoxifying enzymes, and the ability to form biofilms, structures that enhance resistance to antifungal agents and impair immune recognition.^6,7^

Despite its medical importance, treatment options for cryptococcosis remain limited to a few antifungal classes, such as polyenes (e.g., amphotericin B), azoles (e.g., fluconazole), and flucytosine. These drugs are often associated with high cost and toxicity, limited availability, and emerging resistance.^8–10^ Although echinocandins represent the most recent class of antifungals, they are ineffective against *Cryptococcus* spp. due to the unique composition of its cell wall.^11,12^

To address the urgent need for new antifungal agents, open-access initiatives like Medicines for Malaria Venture (MMV), have made diverse compound libraries available to the research community.^13^ These libraries include molecules with established pharmacokinetics and safety profiles, many of which are candidates for drug repurposing. In this study, we screened the 240 compounds from the MMV Global Health Priority Box (GHPB)^14^ for antifungal activity against *C. neoformans* and *C. gattii*, aiming to identify compounds with anti-*Cryptococcus* activity and to investigate their potential as innovative tools for cryptococcosis control. Depending on their pharmacological profile, compounds were further evaluated for either therapeutic development or environmental control of fungal reservoirs.

## 2 Results

### 2.1 The GHPB Screening Identifies Flufenerim as a Hit Compound

The initial antifungal screening of compounds from the GHPB identified flufenerim, an insecticide originally developed for agricultural use, as the only molecule among the 240 compounds present in the GHPB that markedly inhibited cryptococcal growth. At a concentration of 1 μM (equivalent to 0.364 μg/mL), flufenerim suppressed the growth of both *C. neoformans* H99 and *C. gattii* L25/01. The absence of visible turbidity in the wells was considered evidence of antifungal activity (Figure S1).

### 2.2 Flufenerim Has Broad Antifungal Activity against *Cryptococcus* Spp

Minimal inhibitory concentration (MIC) assays were conducted using the broth microdilution method, with Flufenerim concentrations ranging from 8 to 0.015 μg/mL. MIC was defined visually as the lowest concentration with 100% inhibition of growth. Among the 15 strains tested, MIC values varied significantly, ranging from 0.03 μg/mL (strain CFP 00454, *C. gattii*) to >8 μg/mL (strain CFP 00714, *C. gattii*), indicating strain-dependent susceptibility. Previous work from our group demonstrated that these strains exhibit susceptibility to the majority of clinically relevant antifungal drugs.^15^ These results are presented in Table S1. MFC determination was carried out by plating 10 μL from wells with no visible growth onto Sabouraud dextrose agar. After 48 h at 35 °C, the minimal fungicidal concentration (MFC) was defined as the lowest concentration with no further colony growth after flufenerim exposure. Flufenerim exhibited fungistatic activity against most strains, with fungicidal activity observed only for *C. gattii* strain CFP 00455. Table 1 presents MIC and MFC data of all strains evaluated in this study.

**Table 1:** Minimum Inhibitory Concentration (MIC) and Minimum Fungicidal Concentration (MFC) Values (μg/mL) for *Cryptococcus Gattii* and *Cryptococcus neoformans* Strains Treated with Flufenerim

Strain	Species	MIC (μg/mL)	MFC (μg/mL)	Activity
CFP 00428	*C. gattii*	0.25	4	Fungistatic
CFP 00435	*C. gattii*	0.06	8	Fungistatic
CFP 00454	*C. gattii*	0.03	>8	Fungistatic
CFP 00455	*C. gattii*	2	2	Fungicidal
CFP 00713	*C. gattii*	0.125	>8	Fungistatic
CFP 00714	*C. gattii*	>8	>8	Fungistatic
L25/01	*C. gattii*	0.125	>8	Fungistatic
CFP 00661	*C. neoformans*	0.125	>8	Fungistatic
CFP 00662	*C. neoformans*	0.25	>8	Fungistatic
CFP 00664	*C. neoformans*	2	>8	Fungistatic
CFP 00737	*C. neoformans*	0.25	>8	Fungistatic
CFP 00826	*C. neoformans*	0.25	>8	Fungistatic
CFP 00831	*C. neoformans*	0.25	>8	Fungistatic
CFP 00832	*C. neoformans*	0.125	>8	Fungistatic
H99	*C. neoformans*	2	>8	Fungistatic

To complement visual MIC data, fungal growth of reference strains was also quantified by optical density (OD) measurements at 530 nm after 48 h of incubation. The sub inhibitory concentration was defined as the lowest concentration reducing OD by ≥ 50% compared to the positive control. For the H99 strain, although MIC was 2 μg/mL, spectrophotometric analysis showed 50% reduction at 0.125 μg/mL, which was herein used as the sub inhibitory MIC. For the L25/01 strain, spectrophotometric analysis showed 50% reduction at 0.062 μg/mL (Figure 1), which was then used as the sub inhibitory MIC for further assays.

**Figure 1:**
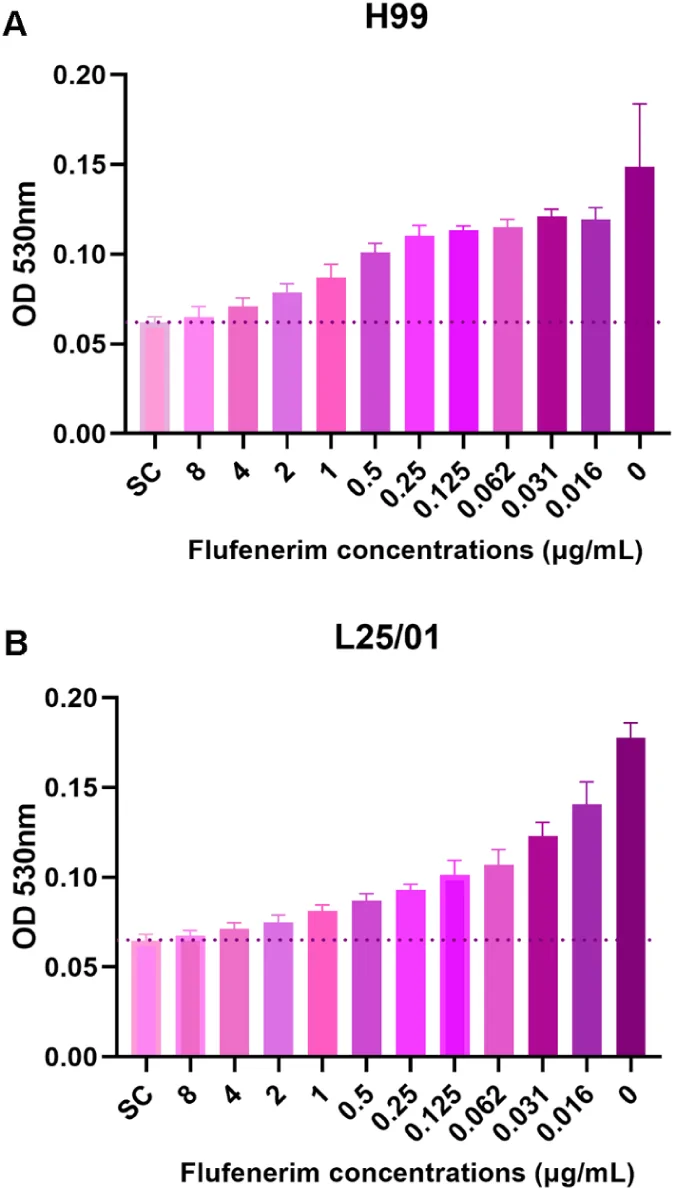
Spectrophotometric analysis of the antifungal activity of the compound against (A) *C. neoformans* H99 and (B) *C. gattii* L25/01 strains. Optical density (OD) at 530 nm was measured after 48 h of incubation. For *C. neoformans* H99, a 50% reduction in growth occurred at 0.125 μg/mL. For *C. gattii* L25/01, this reduction occurred at 0.062 μg/mL. Bars represent the mean and standard deviations of at least four independent experiments performed in duplicate. The dotted line represents the baseline OD where there is no fungal growth. MC: Medium control (RPMI 1640 medium without *Cryptococcus* and without flufenerim).

### 2.3 Flufenerim Impacts Some *Cryptococcus* Spp. Virulence Factors

Treatment with sub inhibitory concentrations of flufenerim induced marked alterations in capsule size in both *Cryptococcus* reference strains. Capsule thickness was significantly reduced in treated cells compared with untreated controls (*P* < 0.0001 for both strains). However, the total cell size (capsule plus cell body) remained unchanged in L25/01 (P = 0.4955), suggesting an increase in cell body size compensated for the reduced capsule thickness. In contrast, H99 exhibited a significant decrease (*P* < 0.0001) in total size following flufenerim treatment (Figure 2).

**Figure 2:**
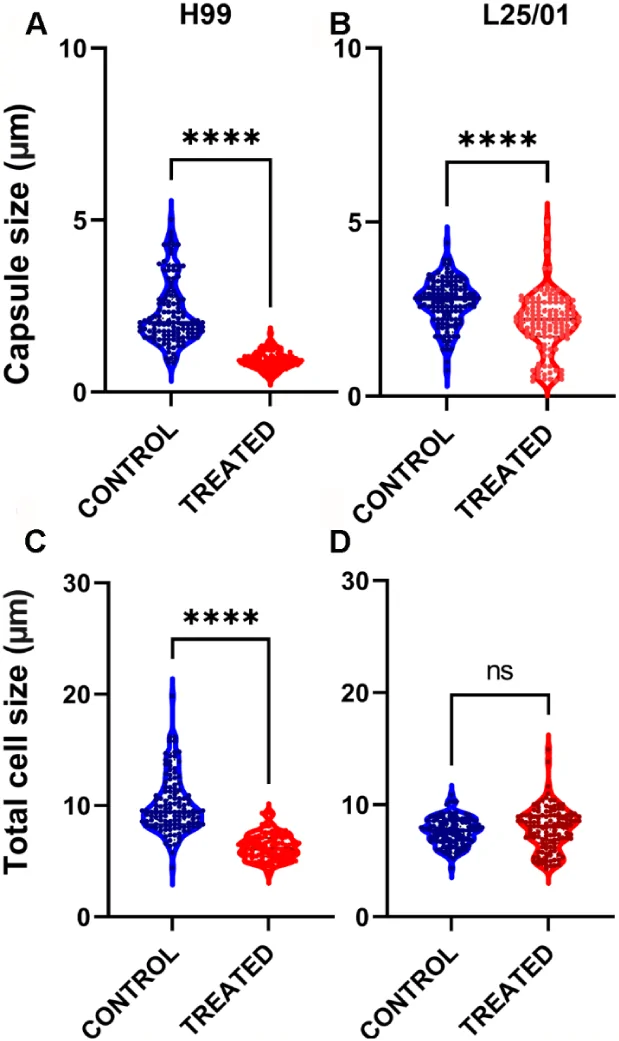
Effect of sub inhibitory concentrations of flufenerim on capsule thickness and cell size in *Cryptococcus* strains H99 and L25/01 (*C. neoformans* and *C. gattii*, respectively). Dots within violins represent individual measurements of the untreated and flufenerim-treated conditions. A total of 100 measurements were made for each species/condition. Black lines inside violins represent the median value for each condition. (A) Capsule size of *C. neoformans* H99. (B) Capsule size of *C. gattii* L25/01. (C) Total cell size of *C. neoformans* H99. (D) Total cell size of *C. gattii* L25/01. **** indicates statistically significant differences (*P* < 0.0001, Mann–Whitney test). ns: not significant.

Melanin production was evaluated in the *C. neoformans* H99 and *C. gattii* L25/01 reference strains grown on Staib’s agar under two conditions: control (no flufenerim) and sub inhibitory concentration of flufenerim (0.125 μg/mL for H99; 0.06 μg/mL for L25/01). Pigmentation was evaluated based on the intensity of brown coloration and the proportion of pigmented cells (Figure 3). Under control conditions, the L25/01 strain exhibited pigmentation levels comparable to those of H99, with the majority of cells appearing fully melanized. Quantitative analysis using ImageJ revealed that flufenerim-treated H99 cells showed a 63.6% reduction in melanization compared to untreated controls, whereas L25/01 cells exhibited a modest 4.4% reduction.

**Figure 3:**
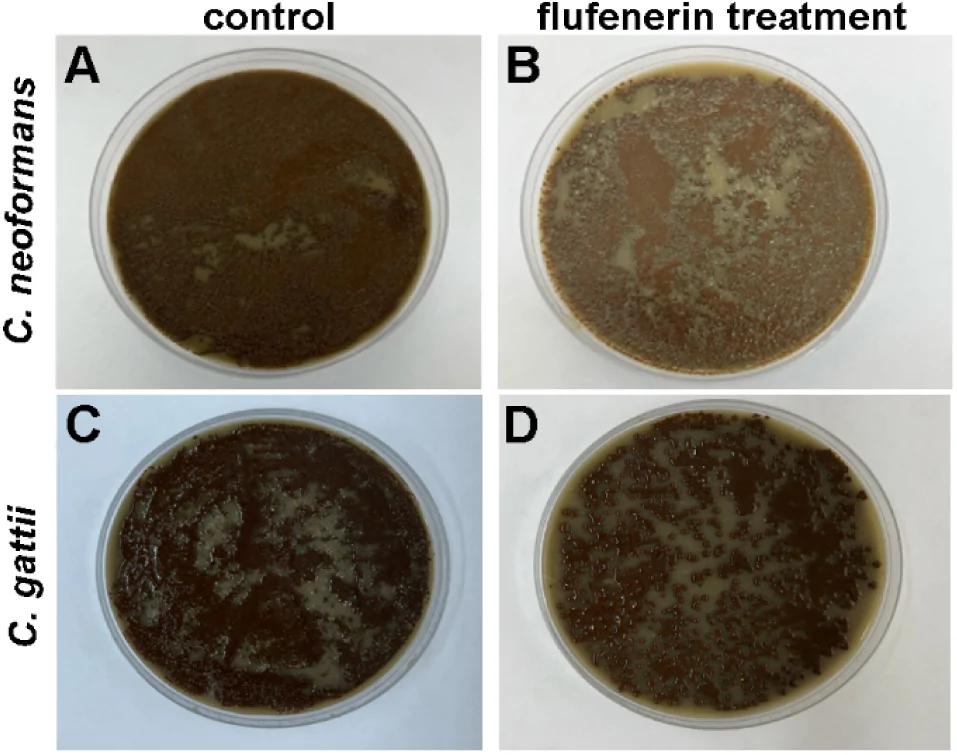
Melanin production in *Cryptococcus neoformans* strain H99 (A and B) and *Cryptococcus gattii* strain L25/01 (C and D) cultivated on Staib’s agar. Strains were incubated in the absence (A and C) and in the presence of flufenerim at sub inhibitory concentrations (B and D). Pigmentation was visually assessed based on the colony color intensity. Images are representative of three independent experiments.

As presented in Figure 4, under control conditions (no flufenerim treatment), both *C. neoformans* H99 and *C. gattii* L25/01 exhibited high urease activity, with mean absorbance values higher than 0.6 OD units measured at 559 nm, confirming their ability to efficiently metabolize urea. Following treatment with flufenerim, no significant reduction in urease activity was observed for *C. neoformans* H99 (P = 0.7797), but a reduction was observed for *C. gattii* L25/01 (*P* < 0.0001). The negative control (medium only), used to ensure assay specificity, showed very low absorbance values (OD 559 nm <0.1), confirming that the measured activity is exclusively due to urease production.

**Figure 4:**
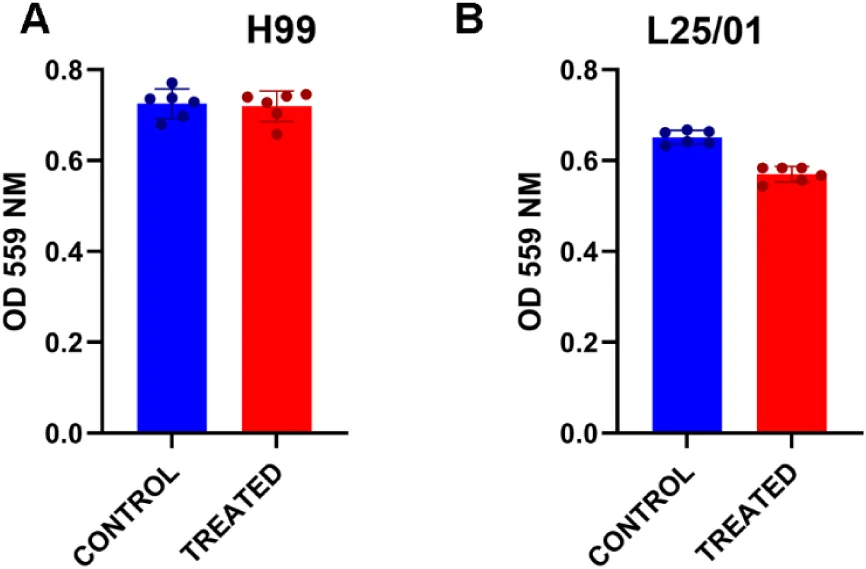
Urease activity of *Cryptococcus neoformans* (H99) and *Cryptococcus gattii* (L25/01) under experimental conditions. Three conditions were evaluated: no flufenerim treatment (control), treatment with sub inhibitory concentrations of flufenerim (treated), and negative control consisting of Christensen’s urea medium without fungal cells (medium). The medium condition remained unchanged for all experiments, indicating that the measured activity is exclusively due to urease production. Dots from control and treatment conditions represent the mean absorbance values, measured at 559 nm, of six independent experiments, reflecting urease enzyme activity levels. Data represent mean ± standard deviation of these measurements. (A) Urease activity of *C. neoformans* H99. (B) Urease activity of *C. gattii* L25/01. No statistical significance was observed among treated and untreated conditions (Student’s *t* test). OD: optical density.

The phospholipase activity indices (Pz) were also evaluated. As shown in Figure 5, no statistically significant differences were observed between the groups, regardless of *Cryptococcus* species tested (*P* > 0.99).

**Figure 5:**
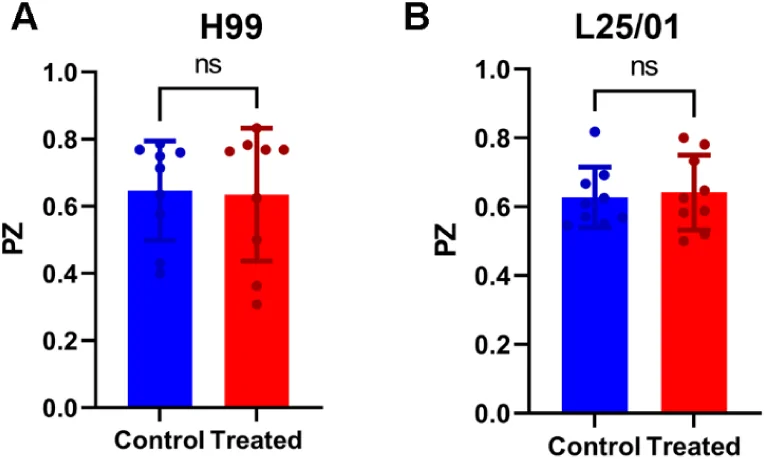
Phospholipase activity indices (Pz) of *Cryptococcus neoformans* H99 (A) and *Cryptococcus gattii* L25/01 (B) under control (untreated) and flufenerim-treated conditions. Dots represent individual measurements of nine experiments. Data represent mean ± standard deviation of four experimental groups. Statistical analysis showed no significant differences between groups (*P* > 0.05, Student’s *t* test).

### 2.4 Flufenerim Has Low Toxicity in Cellular and Invertebrate Models

Cytotoxicity assays across all tested cell lines indicated that flufenerim did not significantly affect the viability of human macrophages (Figure 6A), mouse macrophages (Figure 6B), or human neuronal cells (Figure 6C). After 24 h of exposure, a concentration of 20 μg/mL of flufenerim did not notably reduce cellular viability. These results suggest that the compound is at least ten times more active against most *Cryptococcus* strains than against mammalian cells.

**Figure 6:**
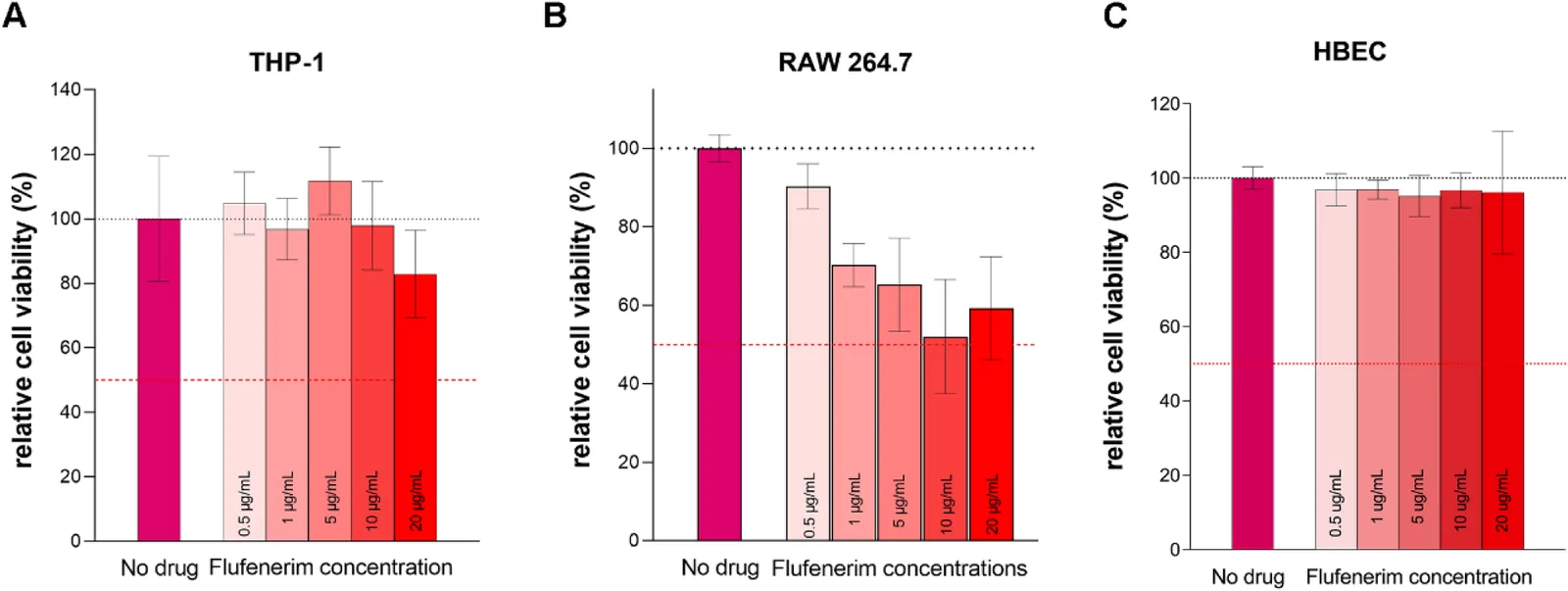
Flufenerim cytotoxicity in three different cell lineages: (A) THP-1, a human monocytic leukemia cell lineage, (B) RAW 264.7, a mice macrophage cell lineage, and (C) HBEC, a human brain endothelial cell lineage. Data represent mean ± standard deviation of at least four replicates. No statistical significance was observed among different flufenerim concentrations (ANOVA test).

Regarding the *in vivo* assay using the alternative invertebrate animal model (Figure 7), no significant mortality was observed in any group, and therefore it was not possible to calculate a lethal dose 50% (LD_50_) for flufenerim under the tested conditions, indicating that the compound produced sub lethal physiological effects in *G. mellonella* rather than acute lethality. Throughout the experimental evaluation, larvae treated with 100 μg/mL exhibited the most pronounced decline in health index, particularly during the first 4 days, followed by a gradual decrease until day 8. Those exposed to 10 μg/mL showed an intermediate pattern, with a slower but progressive decline. Control groups maintained relatively stable health indices, with minor variations likely related to natural aging. These observations are supported by the survival curves and the tabulated health index scores, which provide a representation of larval health dynamics under each treatment condition.

**Figure 7:**
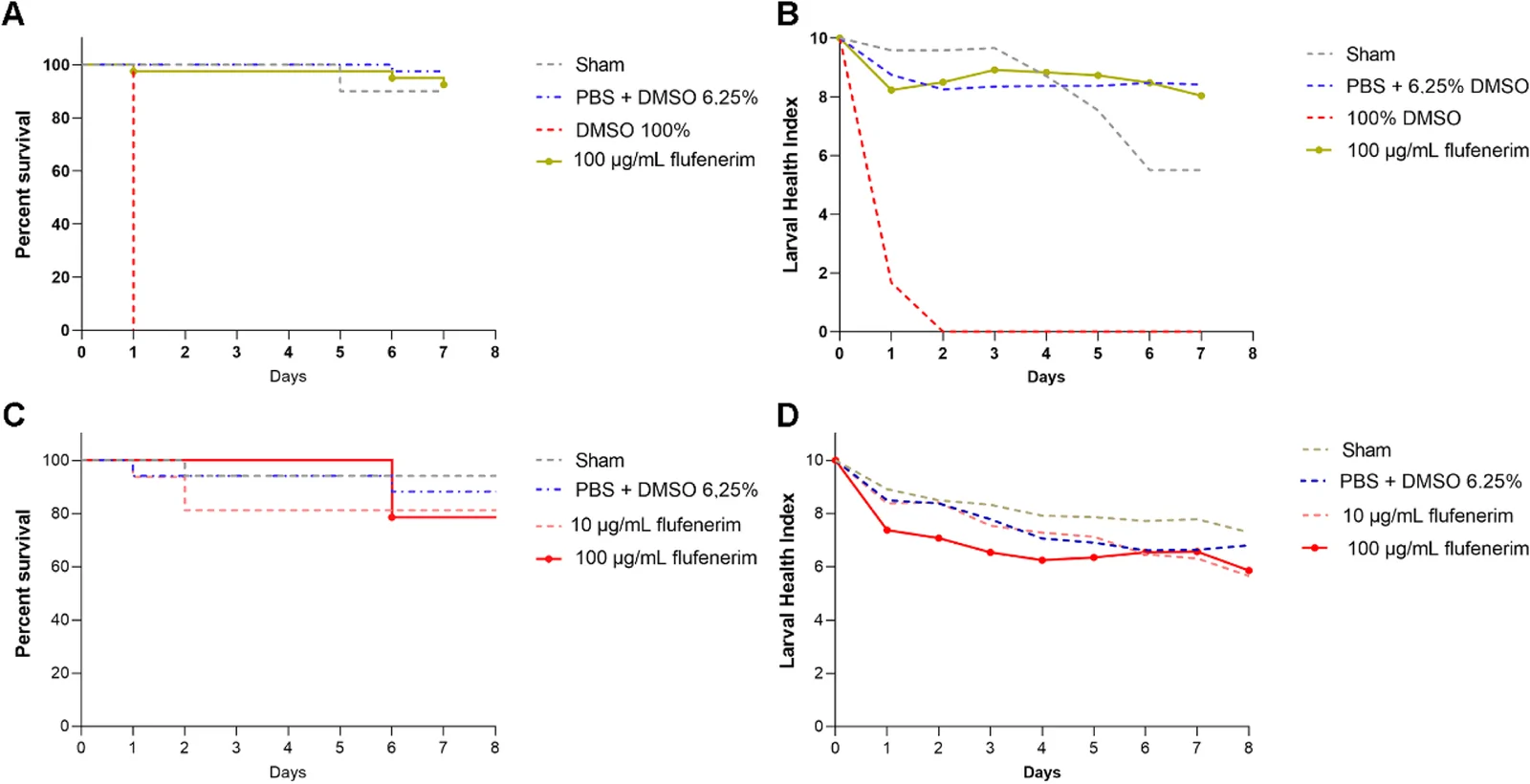
Toxicity assessment of flufenerim in the *Galleria mellonella* model over an 8-day period. Two exposure approaches were evaluated: injection of flufenerim at 100 μg/mL (A, B) and topical exposure by immersion at 10 μg/mL or 100 μg/mL (C, D). Sham and PBS + DMSO groups were included as controls (*n* = 17 larvae per group). Larval survival (A, C) and health index (B, D) were recorded daily based on activity, cocoon formation, melanization, survival, and physical appearance. Data represent the mean of three independent experiments. No significant mortality was observed in any experimental group (Mantel-Cox test).

### 2.5 Flufenerim Does Not Have a Favorable Toxicological Profile

ADMETlab 3.0 predictions indicated that flufenerim exhibits good absorption profile and passes the Lipinski Rule. It penetrates the blood brain barrier, its metabolism is probably hepatic, associated with some cytochrome P450 enzymes, and has a moderate plasma clearance. However, multiple organ toxicities were predicted, including hepatotoxicity, nephrotoxicity, ototoxicity, respiratory, neurotoxicity, and genotoxicity. The main ADMET features are illustrated in Figure 8 and their complete description in Table S2.

**Figure 8:**
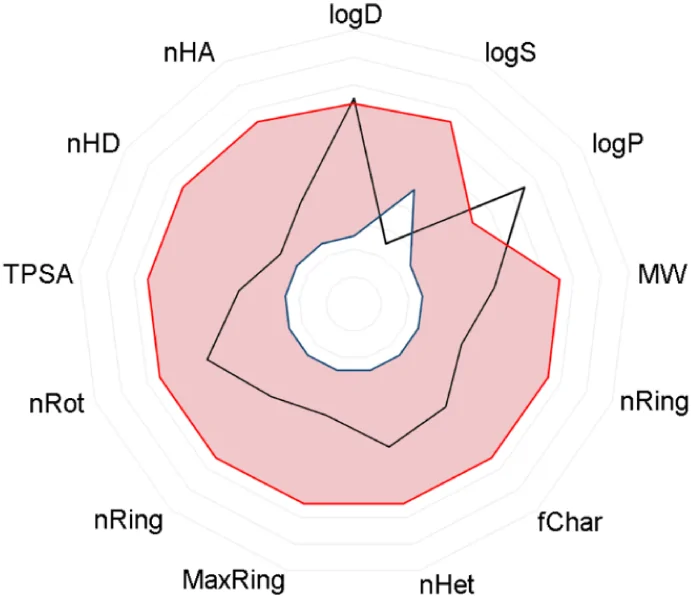
Radar plot of predicted ADMET properties for flufenerim using ADMETlab 3.0. The red area indicates optimal ranges for drug-like compounds, and the black line represents flufenerim values. Most parameters fall within or near optimal ranges. logD: The logarithm of the n-octanol/water distribution coefficient; logS: The logarithm of aqueous solubility value; logP: The logarithm of the n-octanol/water distribution coefficients at pH = 7.4; MW: molecular weight; nRig: Number of rigid bonds; fChar: Formal charge; nHet: Number of heteroatoms; MaxRing: Number of atoms in the biggest ring; nRing: number of rings; nRot: Number of rotatable bonds; TPSA: Topological Polar Surface Area; nHD: Number of hydrogen bond donors; nHA: Number of hydrogen bond acceptors.

### 2.6 Flufenerim Affects the Growth of *C. neoformans* in Contaminated Tree Hollow Samples

Environmental samples spiked with *C. neoformans* H99 were plated on Staib’s agar and incubated at 25 °C for 3 days. The growth of other fungi naturally present in the tree hollow samples prevented precise colony counting of *C. neoformans*. Nevertheless, in the untreated control, numerous brown colonies morphologically consistent with *C. neoformans* were visually observed (more than 3,000 CFU/mL). Similar observations were made in the DMSO-treated control plates, confirming that the solvent, at the tested concentration, did not affect fungal growth. In contrast, plates treated with flufenerim at both MIC and sub inhibitory concentrations showed an apparent visual decrease in the number of brown colonies (less than 1,000 CFU/mL), which appeared more pronounced at the MIC. This qualitative pattern was consistently observed across all plate series. Interestingly, other environmental fungi did not appear to be affected under the same conditions (Figure 9).

**Figure 9:**
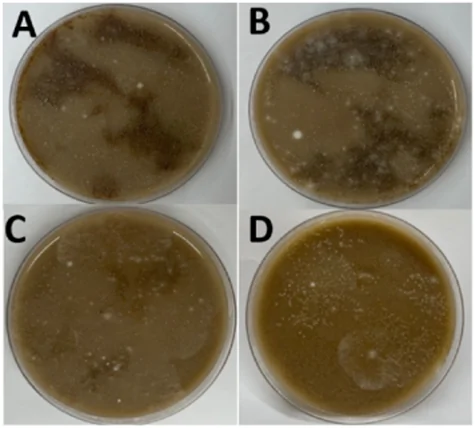
Growth of *Cryptococcus neoformans* H99 on Staib’s agar following treatment of environmental samples with flufenerim. Plates were inoculated with 100 μL of supernatant from environmental samples artificially spiked with H99 (2 × 10^4^ cells/mL) and incubated at 25 °C for 3 days. Brown, yeast-like colonies indicate H99 growth. Experimental conditions included: (A) untreated spiked environmental sample, (B) vehicle control (6.25% DMSO), (C) half-sub inhibitoy dose flufenerim (0.125 μg/mL), and (D) flufenerim minimal inhibitory concentration (2 μg/mL). Images are representative of ten independent plates per condition.

## 3 Discussion

Recently, open-access drug libraries have emerged as valuable tools for identifying repurposing candidates in fungal diseases.^16–19^ Among these resources, the GHPB is particularly relevant, as it aligns with World Health Organization priorities for tackling antimicrobial resistance and neglected tropical diseases.^14^ This library has already contributed to the discovery of compounds with potential activity against emerging and neglected pathogens,^20–22^ including fungal agents that cause mycetoma.^23^ Beyond therapeutic applications, the GHPB has also been explored for control strategies, such as the identification of new disinfectants active against *Candidozyma auris*.^24^ The identification of flufenerim as the sole compound from the GHPB exhibiting antifungal activity against *C. neoformans* and *C. gattii* underscores the value of drug repurposing strategies, particularly when leveraging open-access chemical libraries. Flufenerim has a core pyrimidine structure and exhibits potent activity against the sweet potato whitefly *Bemisia tabaci*, the green peach aphid *Myzus persicae*, and the African cotton leaf worm *Spodoptera littoralis*.^25^

Flufenerim demonstrated potent fungal growth inhibition at low concentrations for most strains. The observed variability in MIC across *Cryptococcus* spp. strains likely reflect intrinsic species- and strain-specific differences in susceptibility. This highlights the importance of evaluating a diverse panel of clinical isolates in future screens. Flufenerim exhibited a predominantly fungistatic profile against *Cryptococcus* spp., consistent with many standard antifungals, such as azoles,^26^ which rely on host immune clearance for optimal efficacy.^27^ Flufenerim has previously demonstrated antifungal activity against the multidrug-resistant fungal pathogen *C. auris*.^24^ This finding suggests that, as with many clinically used antifungals,^28^ flufenerim may exhibit activity across a broader spectrum of pathogenic fungi rather than being restricted to a single species.

Compounds that target fungal virulence determinants rather than exert direct fungicidal activity represent an alternative therapeutic strategy. By attenuating pathogenicity instead of eliminating the organism, these approaches may facilitate host immune clearance while reducing the selective pressure that drives antifungal resistance.^27^ Phenotypic assays revealed a significant and reproducible reduction in cryptococcal capsule thickness at sub inhibitory concentrations of flufenerim. Given its essential role in evasion of host immune responses,^29^ this finding suggests that flufenerim could sensitize *Cryptococcus* cells to phagocytosis and oxidative killing. Interestingly, the compound did not affect melanin, urease, or phospholipase production in both species simultaneously, indicating a possible impact on capsule-related pathways rather than broad virulence suppression, an avenue that warrants future mechanistic studies, such as transcriptomic profiling or targeted genetic knockouts, to elucidate the precise molecular targets. In addition, the differences in melanin observations may reflect cells that are still undergoing melanization or a lower proportion of pigmented cells in the observed field, which needs further clarification.

Flufenerim was first described as a synthetic insecticide belonging to the pyrimidinamine group, with efficacy against certain insects.^25^ In the present study, toxicity assays demonstrated low cytotoxicity toward the three mammalian cell lines tested, suggesting a favorable preliminary safety profile. However, previous evidence of cytotoxicity against human hepatoma HepG2 cells^22^ indicates that its toxicity may be context- or cell-type dependent, warranting further investigation. The *G. mellonella* model provided additional insights, as treatment with flufenerim at high concentrations induced sub lethal physiological effects without causing significant mortality. It should be noted that, assuming homogeneous distribution throughout the larval body, the estimated internal concentration of flufenerim following treatment would be approximately 3.33 μg/mL. This value exceeds the MICs observed for most *Cryptococcus* strains evaluated in the present study. The absence of acute toxicity in this invertebrate host may be partially explained by the compound’s limited insecticidal spectrum, since it has been reported to be ineffective against certain insects such as *Frankliniella occidentalis* and *Thrips tabaci*.^25^ Nevertheless, further studies are required to determine whether the sub lethal effects observed in the invertebrate model translate to vertebrate systems and to establish dose ranges that balance antifungal efficacy with host safety.

To further evaluate the safety profile of flufenerim, we performed an ADMET analysis to characterize its predicted pharmacokinetics and toxicity.^30^ The results indicated several favorable parameters, including compliance with Lipinski’s rules, good absorption, and blood–brain barrier penetration, traits that would be advantageous for developing a novel cryptococcosis therapy.^31^ However, the prediction of multiple organ toxicities discouraged further consideration of flufenerim for systemic drug repurposing. Given its original development as an insecticide, we hypothesized that flufenerim could instead be applied in the environment to decontaminate areas known to harbor *Cryptococcus* spp., a field with only limited exploration.^32,33^ A similar strategy has been proposed for controlling *C. auris* in healthcare environments.^24^

Supporting this hypothesis, flufenerim reduced the recovery of *C. neoformans* from tree-hollow samples, providing a preliminary proof-of-concept for environmental application. Nevertheless, these observations were qualitative; quantitative culture-based assays or viability-based molecular approaches to accurately quantify fungal reduction such as PMA-qPCR or reverse transcription qPCR targeting RNA may provide more reliable estimates of viable *Cryptococcus* populations. Moreover, because capsule production occurs in environmental conditions,^34^ its impairment by flufenerim may potentially influence the virulence of *Cryptococcus* cells that persist after exposure. Such modulation could render residual yeasts more susceptible to immune recognition and clearance, thereby potentially affecting infection establishment.

These findings identify flufenerim as a novel anti-*Cryptococcus* compound and provide preliminary evidence supporting its potential for reducing *Cryptococcus* recovery from environmental substrates. The ecology of *Cryptococcus* spp. is highly complex, with fungal survival and dissemination depending on numerous ecological factors and occurring across diverse environmental niches, including soil, tree hollows, decaying wood, ants, and bird excreta.^35^ Therefore, additional investigations are required to determine whether similar effects would be observed in other natural reservoirs and ecological settings.

In addition, we have selected *C. neoformans* for this experiment because it is the most widely distributed environmental agent of cryptococcosis worldwide, whereas *C. gattii* is geographically restricted to specific endemic regions. Nevertheless, the ecology of the cryptococcosis agents is largely comparable, and both species exhibited similar susceptibility profiles and phenotypic responses to flufenerim throughout the study. Therefore, we believe that the findings obtained with *C. neoformans* are likely to be representative of *C. gattii* as well, although future studies directly evaluating both species under environmental conditions would be valuable to confirm this assumption.

The mechanism underlying the antifungal activity of flufenerim remains unknown and warrants further investigation. The predominantly fungistatic profile observed for most strains further supports the hypothesis that flufenerim acts by impairing cellular functions required for growth and virulence rather than causing rapid cell death, such as glucuronoxylomannan biosynthesis. Future studies employing multiomics and electron microscopy-based approaches will be necessary to identify the molecular targets of flufenerim and determine whether its antifungal activity is mediated through capsule-specific pathways or broader effects on cryptococcal physiology. In addition, they should evaluate flufenerim interaction with existing antifungals, investigate the potential for resistance development, and determine its efficacy and ecological safety under diverse environmental conditions.

## 4 Conclusions

In summary, this study identified flufenerim as the sole active compound from the GHPB with antifungal activity against *C. neoformans* and *C. gattii*. Despite its original development as a pyrimidinamine insecticide, flufenerim demonstrated consistent antifungal effects *in vitro*, including inhibition of growth at low concentrations and modulation of capsule size, a major virulence determinant of *Cryptococcus*. While ADMET predictions suggested favorable pharmacokinetic properties, multiple organ toxicities strongly discourage its repurposing as a therapeutic drug. Nevertheless, the compound showed promising activity under ecologically relevant conditions, reducing *C. neoformans* growth in an environmental sample without apparent impact on other fungi. Considering that capsule production also occurs in the environment, its inhibition by flufenerim may further decrease the virulence of residual yeasts, potentially facilitating immune clearance. Together, these findings suggest that flufenerim represents a useful experimental tool for investigating environmental control strategies against *Cryptococcus*. Nevertheless, its ecological impact, selectivity, and effectiveness across diverse environmental reservoirs remain to be established.

## 5 Materials and Methods

### 5.1 Strains

The reference strains *C. neoformans* H99 (ATCC 208821) and *C. gattii* L25/01 were employed in all experiments. Additionally, seven clinical isolates of *C. neoformans* and seven of *C. gattii*, all isolated from cerebrospinal fluids from human patients, were included in selected assays, as detailed in the corresponding sections. Information regarding these strains is provided in Table S1. These strains were used in a previous work testing antifungal susceptibility of *Cryptococcus* spp. against five antifungal drugs (amphotericin B, 5-flucytosine, fluconazole, voriconazole, and itraconazole) by three different methods.^15^

### 5.2 Compound Library

The Global Health Priority Box, provided by MMV in collaboration with partner institutions, is a curated collection of 240 compounds distributed in three plates. These molecules represent diverse stages of drug discovery and were selected based on their previously demonstrated activity against pathogens or vectors of medical importance. The library includes 80 compounds active against drug-resistant malaria, 80 molecules sourced from pharmaceutical collections with potential application in neglected and emerging infectious diseases, and 80 compounds tested for activity against insect vectors relevant to disease transmission. Compounds were supplied as lyophilized powder, each corresponding to a 10 mM stock solution when reconstituted in 10 μL of dimethyl sulfoxide (DMSO). The reconstituted plates served as mother plates from which multiple experimental plates were prepared when necessary. Following the initial DMSO dilution, further dilutions in the appropriate culture medium were prepared to minimize DMSO concentration during experimental procedures.

### 5.3 Screening of the GHPB

The representative strains of *C. neoformans* H99 and *C. gattii* L25/01 were cultured in Sabouraud Dextrose Agar (Becton, Dickinson and Company, Sparks, MD, USA) at 37 °C for 24 h. Then, yeasts were suspended in RPMI 1640 medium (Sigma-Aldrich, St. Louis, MO, EUA) supplemented with 2% glucose (Sigma-Aldrich) at a concentration of 2 × 10^5^ cells/mL and exposed to each compound of the GHPB at a final concentration of 1 μM in a final volume of 200 μL in 96-well plates, as recommended by the manufacturer.^13,14^ Controls included: (i) 200 μL of RPMI 1640 medium without fungal cells as a negative control; and (ii) 200 μL RPMI + 1% DMSO containing fungal cells as the growth control. Plates were incubated at 37 °C for 48 h, and fungal growth inhibition was evaluated by visual inspection. Wells without fungal growth, as observed in the negative control, were considered as hits. The selected hit compounds were subsequently obtained from MMV as pure powders at no cost. Stock solutions were prepared at a concentration of 1.6 mg/mL.

### 5.4 Determination of Minimum Inhibitory Concentration (MIC)

MIC values were determined using the broth microdilution method according to the EUCAST guidelines, with minor modifications. Serial 2-fold dilutions of the selected compounds were prepared in RPMI 1640 medium supplemented with 2% glucose, over a concentration range of 0.015 to 8 μg/mL. Fungal inocula were standardized to 0.5–2 × 10^5^ cells/mL, and plates were incubated at 37 °C for 48h. The visual MIC was defined as the lowest concentration at which complete inhibition of visible fungal growth occurred. To complement the visual assessment, fungal proliferation was also quantified spectrophotometrically by measuring the optical density (OD) at 530 nm. To evaluate the antifungal susceptibility of hit compounds that are not described in the EUCAST protocol, their MIC was defined as the lowest concentration at which OD was reduced by at least 90% relative to the untreated growth control. Additionally, a sub inhibitory concentration was defined as the lowest compound concentration that produced a ≥ 50% reduction in OD_530_ relative to the control. This sub inhibitory concentration was subsequently used in phenotypic assays to investigate potential alterations in key virulence factors.

### 5.5 Determination of Minimum Fungicidal Concentration (MFC)

To determine the MFC, 10 μL aliquots were taken from wells showing no visible growth in the previous MIC assays and plated on Sabouraud dextrose agar. Plates were incubated at 37 °C for 48 h. The MFC was defined as the lowest compound concentration at which no fungal colony growth was observed. The MFC/MIC ratio was subsequently calculated to classify the compound’s effect, with values ≤ 2 indicating fungicidal activity and values >2 indicating a fungistatic profile.^17^

### 5.6 Assessment of Virulence Factors

To assess the impact of hit compounds on cryptococcal virulence traits, assays were conducted under specific experimental conditions. All assays were performed using sub inhibitory concentrations of the selected hit compounds. The following virulence factors were evaluated: (i) capsule formation; (ii) melanin production; (iii) urease activity; and (iv) phospholipase activity.

Virulence assays were performed using the reference strains *Cryptococcus neoformans* H99 and *Cryptococcus gattii* L25/01 due to their extensive characterization and widespread use in experimental virulence studies, allowing reproducibility and comparison with previously reported data. Prior to treatment, strains were subcultured on Sabouraud dextrose agar and incubated at 37 °C for 24 h. Colonies were then suspended in sterile 0.9% saline solution and adjusted to approximately 10^6^ cells/mL. Subsequently, 9 mL of liquid Sabouraud medium was mixed with 1 mL of the standardized cell suspension, and 2.5 mL aliquots were transferred into sterile 15 mL Falcon tubes. Hit compounds were added at their respective sub inhibitory concentrations. This concentration was selected for virulence assays because higher concentrations could significantly reduce cell viability, thereby complicating the interpretation of phenotypic assays that depend on metabolically active fungal cells.^35^ After treatment, cells were incubated under specific experimental conditions prior to the evaluation of each virulence factor, as described below.

#### 5.6.1 Morphometric Analyses

Capsule formation was evaluated after exposure to the hit compound at a sub inhibitory concentration. Following incubation in Sabouraud medium at 37 °C for 48 h in the presence of the compounds, cells were collected and transferred to capsule-inducing minimal medium,^36^ composed of 15 mM dextrose, 10 mM MgSO_4_, 29.4 mM KH_2_PO_4_, 13 mM glycine, and 3 μM thiamine (pH 5.5), sterilized by filtration. Cells were incubated under capsule-inducing conditions prior to analysis. To assess capsule size, cells were centrifuged at 5,000 rpm for 10 min, negatively stained with India ink, and visualized using an AXIO Lab.A1 optical microscope (ZEISS, Jena, Germany). Capsule thickness, cell body diameter, and total cell diameter were quantified in at least 100 cells using ImageJ software version 1.8.0 g.^37^ Digital micrographs were first calibrated using the microscope scale bar. For each cell, the maximum diameter of the cell body and the maximum diameter including the capsule were measured using the straight-line tool. Capsule thickness was calculated as half the difference between the total cell diameter and the cell body diameter, according to the formula: capsule thickness = (total diameter – cell body diameter)/2. At least 100 randomly selected cells were measured for each experimental condition.^38^

#### 5.6.2 Melanin Production

Melanin production was evaluated in the reference strains *C. neoformans* H99 and *C. gattii* L25/01, after treatment with the hit compounds as described above. Following treatment, cultures were centrifuged, and the resulting cell pellets were resuspended in 0.9% sterile saline. The suspensions were diluted 1:1,000 and vortexed to ensure homogenization. Subsequently, 100 μL of the diluted suspension were plated on Staib’s agar and evenly spread across the plate surface. The medium contained (per liter): 50 g *Guizotia abyssinica* (Niger seed) extract, 10 g glucose, 1 g KH_2_PO_4_, 1 g creatinine, and 20 g agar, dissolved in distilled water to a final volume of 1 L. Untreated controls were processed in parallel. Plates were incubated at 25 °C for up to 4 days, and melanization was assessed by direct visual inspection and through pixel intensity of plate photographs using the ImageJ software.

#### 5.6.3 Phospholipase Activity Assay

After 48 h of treatment, cultures of the reference strains *C. neoformans* H99 and *C. gattii* L25/01 treated or not with hit compounds were centrifuged at 2,000 g for 10 min, and the pellets were resuspended in sterile 0.9% saline. Then, 10 μL of the cell suspension were directly spotted onto the surface of egg yolk agar plates prepared with Sabouraud dextrose agar supplemented with 1 M NaCl, 5 mM CaCl_2_, and 8% sterile egg yolk emulsion (pH 7.0), as previously described by Price MF. Inoculations were performed in triplicate, and plates were incubated at 37 °C for up to 7 days. Enzymatic activity was assessed by observing precipitation zones surrounding the colonies. The colony diameter (a) and the total diameter including the precipitation zone (b) were measured, and the phospholipase activity index (Pz) was calculated as a/b. Phospholipase activity was classified according to Pz values as follows: Pz = 1.0 was considered negative; 0.64 < Pz < 1.0 indicated low activity; 0.50 ≤ Pz ≤ 0.64 indicated moderate activity; and Pz < 0.50 indicated high phospholipase activity, as previously described.^38^

#### 5.6.4 Urease Activity Assay

Urease activity was evaluated in *C. neoformans* H99 and *C. gattii* L25/01 after exposure to the hit compound at a sub inhibitory concentration, as previously described. Assays were performed in a final volume of 2 mL of Christensen’s urea broth, incubated at 37 °C with agitation (150 rpm) for 48 h. Following incubation, cultures were centrifuged at 2,000 g for 10 min, and 100 μL of the supernatant were transferred to flat-bottom 96-well polystyrene plates (Corning, Tewksbury, USA). Urease activity was indirectly quantified spectrophotometrically by measuring absorbance at 559 nm.^39^

### 5.7 Cytotoxicity Assays

Cytotoxicity of hit compounds was evaluated in three cell lines: THP-1, RAW 264.7, and human brain endothelial cells (HBEC). THP-1, a human monocytic leukemia cell line, was cultured in nontreated plastic bottles containing RPMI 1640 (Nova Biotecnologia, BR) supplemented with 10% heat-inactivated fetal bovine serum (FBS—LGC Biotecnologia, BR), and 1% penicillin/streptomycin (Nova Biotecnologia, BR). Before the assay, THP-1 monocytes were differentiated into macrophages by incubation with 100 ng/mL of phorbol 12-myristate 13-acetate (PMA—GibcoTM) for 72 h, as described elsewhere with some modifications.^40^

As for RAW 264.7, a mice macrophage cell line, the cells were cultured in treated plastic bottles containing DMEM (Nova Biotecnologia, BR) supplemented with 5% FBS and 1% penicillin/streptomycin. Both cell lines were cultivated in nonphenol red media and placed in a humidified incubator at 37 °C and 5% CO_2_. The RAW 264.7 cell line was kindly provided by Dr. Luciana Thomaz from the Laboratory of *Leishmania* Cellular Immunobiology at University of Sao Paulo, Brazil. After acquiring exponential growth conditions, the bottles were placed in ice and, after 5 min, the cells were detached using a cell scraper. Then, they were counted, the viability checked by Trypan Blue staining (LGC Biotecnologia, São Paulo, Brazil) and transferred to separate 96-well plates in a density of 5 × 10^4^ cells/well. Cell were then incubated with growth media with different concentrations of the hit compound (0.5 – 20 μg/mL). For the cytotoxicity assay with these two cell lines, the 3-(4,5-dimethylthiazol-2-yl)-2,5-diphenyltetrazolium bromide (MTT—Thermo Fisher Scientific Corporation, USA) method was performed according to the manufacturer’s instructions. The cells were in contact with the compounds for 24, 48, and 72 h. After each time, 5 mg/mL of MTT was added to the wells and incubated again for 3 h at 37 °C and 5% CO_2_. Then, the plates were centrifuged at 4.000 rpm, 10 min and the formazan crystal was solubilized in 100% DMSO. The absorbance was acquired in 540 nm by BioTek Epoch Microplate Spectrophotometer. Background values of the wells without cells were subtracted from values of tested wells. The untreated cells were used as reference for 100% relative viability. Each condition of this assay was performed in three replicates.

To evaluate the cytotoxicity of compound in HBEC lineage, a viability assay was performed using the redox dye resazurin (Alamar Blue, Thermo Fisher). Initially, the cells were plated in 96-well plates at a concentration of 5 × 10^5^ cells/mL, using DMEM supplemented with 10% fetal bovine serum (FBS) and sodium pyruvate at 100 μg/mL as the culture medium. Cells were kept at 37 °C under 5% CO2 for 24 h. On the following day, the cells were expanded to 1 × 10^6^ cells/mL. After removing the supernatant, fresh DMEM supplemented with 10% FBS and 100 μg/mL sodium pyruvate was added. The cells were then treated with compound at concentrations ranging from 0.5–20 μg/mL, diluted in culture medium containing DMSO as the vehicle. Controls included cells exposed to relative concentrations of DMSO as vehicles for drug solubilization. After 24 h of incubation with the compound, 25 μL of Alamar Blue solution was added to each well, followed by a further 4 h incubation at 37 °C in the dark. Absorbances were measured at 570 nm in a spectrophotometer (SpectraMax, Molecular Devices), and untreated cells were used as a reference for 100% relative viability. Each experimental condition was performed in quadruplicate.

### 5.8 Toxicity in the Animal Model *Galleria mellonella*

The alternative invertebrate model, *Galleria mellonella*, was employed to assess the toxicity of the hit compound. Two complementary approaches were used: (i) the traditional assay, in which larvae were directly inoculated with the compounds; and (ii) a surface exposure assay, designed to evaluate potential toxicity upon contact with the larval cuticle. All phases of *G. mellonella* were maintained in nontoxic plastic boxes at 28.5 °C in the dark, with a cleaning routine performed every 2 days. The larvae were fed with dehydrated pollen and beeswax (Apiários Seiva das Flores, São José do Rio Preto, SP, Brazil). For this experiment, 16 larvae per group were selected by their weight and appearance, having approximately 250 mg, and the absence of dark coloration or pigmentation. One day before the experiment, the selected larvae were randomly put in 120 × 15 mm plate dishes with paper filters and without food, by their respective groups and incubated at 37 °C. On the experiment day, each larvae was carefully cleaned with sterile cotton with 70% ethanol and received a 10 μL injection of the compound at a 100 μg/mL on the last proleg with the aid of a Hamilton syringe. Controls were made for the untouched larvae group (Sham), for the injection of vehicle (PBS+6.25% DMSO), and 100% DMSO.

For the surface exposure assay, healthy final-instar *G. mellonella* larvae were randomly assigned to four experimental groups (n = 17 per group): sham group (handled but not exposed to solvent or compound), vehicle control group (PBS + 6.25% DMSO), hit compound at 10 μg/mL, and hit compound at 100 μg/mL. Treatments were performed by pipetting 10 μL of each solution onto the inner lid of Petri dishes, followed by gently rolling individual larvae over the droplet for 5 s to ensure uniform surface contact. Larvae were then transferred to appropriate containers and maintained at 25 °C. Survival assessments were performed daily for 8 days in both assays. Larvae were considered dead if they showed no response to touch and exhibited cuticle darkening.

In addition to the two previously described assays, larval condition was assessed using a health index.^41^ The following parameters were recorded: (i) activity, determined by spontaneous movement and response to tactile stimulation; (ii) cocoon formation, categorized as absent, partial, or complete conversion to pupa; (iii) melanization, evaluated by the extent and distribution of dark pigmentation; (iv) survival, recorded as alive or dead; and (v) physical aspects, including the presence or absence of deformities. Scores for each parameter were assigned and summed to generate the overall health index, with higher values indicating healthier larvae.

### 5.9 Computational Prediction of ADMET Properties

To further assess the feasibility of developing the identified hit compounds into new therapeutic options for cryptococcosis, their absorption, distribution, metabolism, excretion, and toxicity (ADMET) properties were predicted using the *in silico* platform ADMETlab 3.0.^30^ For this analysis, the SMILES structures of the hit compounds were provided as input to the computational model.

### 5.10 Proof of Concept for Environmental Compound Use

One gram of an environmental sample collected from a *Cassia grandis* hollow was macerated using a mortar and pestle under sterile conditions. Previous studies have demonstrated the presence of *C. neoformans* in this tree species, indicating that it serves as an environmental reservoir for the fungus.^42^ The sample was artificially inoculated with a suspension of the *C. neoformans* H99 strain at a concentration of 2 × 10^4^ cells/mL. The suspension was then transferred to a 125 mL Erlenmeyer flask containing 50 mL of 0.9% saline solution supplemented with 400 mg/L chloramphenicol. The mixture was vortexed for 5 min, and subsequently allowed to rest for 30 min to promote sediment-supernatant separation. The supernatant was collected using a 1 mL pipet and distributed into four Falcon tubes under the following conditions: positive control (no treatment), 1.25% DMSO diluted in PBS, hit compound at sub inhibitory concentration, and at its MIC. Tubes were incubated with moderate shaking at 25 °C for 48 h. After incubation, 100 μL of each condition was plated on 10 Staib’s Agar (pH 5.5) plates using a Drigalski loop. Plates were observed up to 4 days, and the presence of brown, yeast-like colonies was considered indicative of *C. neoformans* growth.

### 5.11 Statistical Analyses

Analyses were performed using GraphPad Prism 9.0 software. The Shapiro-Wilk test was used to check data normality. The Mann–Whitney test was used to compare medians of two independent groups with a non-normal distribution. Student’s *t* test or ANOVA were used to compare groups with normal distribution. Survival curves were evaluated using the Mantel-Cox test. *P* < 0.05 was considered significant.

## Supplementary Material



## Notes

Figure S1: inhibition of *C. neoformans* (H99) and *C. gattii* (L25/01) growth by 1 μM Flufenerim, one of the 240 compounds from the GHPB. (A) *C. neoformans* H99 and (B) *C. gattii* L25/01. The plates contained RPMI 1640 medium supplemented with 2% glucose. Column 1 represented the negative control (medium only) and column 12 represented the growth control (compound-free medium and yeast cells). Columns 2 to 11 contained a different chemical compound per well. Plates were incubated for 48 h at 37 °C. The F11 well, indicated by the red circle in both plates, showed no visible turbidity, indicating effective antifungal activity (TIF); Table S1: strains used in the present study; Table S2: ADMET properties of Flufenerim (PDF)
