# Supplementary material for: Repurposing the Insecticide Flufenerim, the Only Anti-Cryptococcus Compound Identified in the MMV Global Health Priority Box, as a Candidate for the Environmental Control of the Cryptococcosis Agents
Source: ACS Omega. 2026 Oct 6;11(41):62717–27. doi: 10.1021/acsomega.6c04207 (PMC13688482; doi:10.1021/acsomega.6c04207)
Supplement: Supplementary file 1 [file ao6c04207_si_001.pdf]

Repurposing the insecticide flufenerim, the only anti-*Cryptococcus* compound identified in the MMV Global Health Priority Box, as a candidate for the environmental control of the cryptococcosis agents

Juliana Godoy[1], Iara Bastos de Andrade[2], Edinaldo Castro de Oliveira[1], Igor Avellar-Moura[3], Kamilla Xavier Gomes[4], Felipe Carvalho da Costa[5], Marcelo Kurgonas de Oliveira[5], Carlos Pelleschi Taborda[5], Allan Jefferson Guimarães[4,6], Rosely Maria Zancopé-Oliveira[1], Marcos Abreu Almeida[4,6], Susana Frases[3,6], Rodrigo Almeida-Paes[1,6*]

[1] Fundação Oswaldo Cruz, Instituto Nacional de Infectologia Evandro Chagas, Laboratório de Micologia, Rio de Janeiro, RJ, Brazil

[2] Universidade Federal de São Paulo, Departamento de Medicina, Divisão de Doenças Infecciosas, Laboratório Especial de Micologia, São Paulo, Brazil

[3] Universidade Federal do Rio de Janeiro, Instituto de Biofísica Carlos Chagas Filho, Laboratório de Biofísica de Fungos, Rio de Janeiro, RJ, Brazil

[4] Universidade Federal Fluminense, Instituto Biomédico, Laboratório de Bioquímica e Imunologia das Micoses, Niterói, RJ, Brazil

[5] Universidade de São Paulo, Instituto de Ciências Biológicas, Departamento de Microbiologia, Laboratório de Fungos Patogênicos Dimórficos, São Paulo, SP, Brazil

[6] Rede Micologia, Fundação de Amparo à Pesquisa do Estado do Rio de Janeiro (FAPERJ), Rio de Janeiro, RJ, Brazil.

*** Corresponding author:** rodrigo.paes@ini.fiocruz.br

Figure S1. Inhibition of *C. neoformans* (H99) and *C. gattii* (L25/01) growth by 1 µM Flufenerim, one of the 240 compounds from the GHPB**.** (A) *C. neoformans* H99 and (B) *C. gattii* L25/01. Plates contain RPMI 1640 medium supplemented with 2% glucose. Column 1 represents the negative control (medium only) and column 12 the growth control (compound-free medium and yeast cells). Columns 2 to 11 contain one different chemical compound per well. Plates were incubated for 48 h at 37 °C. The F11 well, indicated by the red circle in both plates, shows no visible turbidity, indicating effective antifungal activity (TIF).

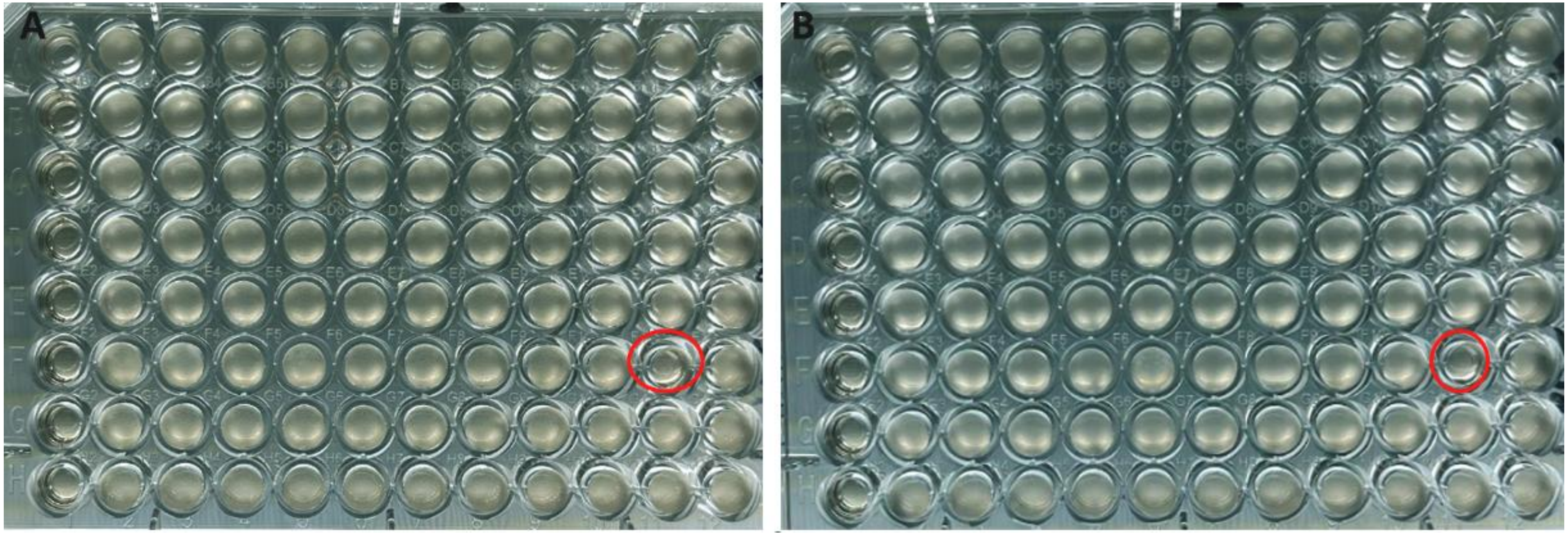

Table S1: Strains used in the present study.

Species	Strain ID	Clinical sample	Origin	Fluconazole MIC	Flucytosine MIC	Amphotericin B MIC
C. neoformans	H99	Cerebrospinal fluid	Reference	2 µg/mL	1 µg/mL	0.25 µg/mL
C. gattii	L25/01	Cerebrospinal fluid	Reference	8 µg/mL	1 µg/mL	0.25 µg/mL
C. gattti	CFP 00428	Cerebrospinal fluid	Rio de Janeiro, Brazil	8 µg/mL	1 µg/mL	0.125 µg/mL
C. gattii	CFP 00435	Cerebrospinal fluid	Rio de Janeiro, Brazil	2 µg/mL	2 µg/mL	0.5 µg/mL
C. gattii	CFP 00454	Cerebrospinal fluid	Roraima, Brazil	2 µg/mL	2 µg/mL	0.125 µg/mL
C. gattii	CFP 00455	Cerebrospinal fluid	Bahia, Brazil	2 µg/mL	2 µg/mL	0.25 µg/mL
C. gattii	CFP 00713	Cerebrospinal fluid	Rio de Janeiro, Brazil	4 µg/mL	1 µg/mL	0.25 µg/mL
C. gattii	CFP 00714	Cerebrospinal fluid	Rio de Janeiro, Brazil	16 µg/mL	2 µg/mL	0.5 µg/mL
C. neoformans	CFP 00661	Cerebrospinal fluid	Rio de Janeiro, Brazil	2 µg/mL	4 µg/mL	1 µg/mL
C. neoformans	CFP 00662	Cerebrospinal fluid	Rio de Janeiro, Brazil	4 µg/mL	2 µg/mL	1 µg/mL
C. neoformans	CFP 00664	Cerebrospinal fluid	Distrito Federal, Brazil	8 µg/mL	2 µg/mL	0.5 µg/mL

Table S2. ADMET properties of Flufenerim

ADMETlab 3.0

CC(F)c1ncnc(NCCc2ccc(OC(F)(F)F)cc2)c1Cl

1. Physicochemical Property

Property	Value	Comment
		Contain hydrogen atoms. Optimal:100~600
		Van der Waals volume
		Density = MW / Volume
		Number of hydrogen bond acceptors. Optimal:0~12
		Number of hydrogen bond donors. Optimal:0~7
		Number of rotatable bonds. Optimal:0~11
		Number of rings. Optimal:0~6
		Number of atoms in the biggest ring. Optimal:0~18
nHet	9.0	Number of heteroatoms. Optimal:1~15
fChar	0.0	Formal charge. Optimal:-4 ~4
nRig	12.0	Number of rigid bonds. Optimal:0~30
Flexibility	0.583	Flexibility = nRot /nRig
Stereo Centers	1.0	Stereo Centers. Optimal: ≤ 2
TPSA	47.04	Topological Polar Surface Area. Optimal:0~140
		The logarithm of aqueous solubility value.
	-5.598<br>4.681	The logarithm of the n-octanol/water distribution coefficients at pH=7.4.<br>The logarithm of the n-octanol/water distribution
	4.08	coefficient.
pka (Acid)	9.178	
pka (Base)	4.296	Acid-base dissociation constant (pKa) value represents the strength of a drug molecule's acidity or basicity.
Melting point	72.929	The predicted melting point of a compound is expressed in degrees Celsius (°C).<br>Melting points below 25°C are classified as liquids, while melting points above 25°C are classified as solids.
Boiling point	332.04	The predicted melting point of a compound is expressed in degrees Celsius (°C).<br>A normal boiling point below 25°C is categorized as a gas.

2. Medicinal Chemistry

Property	Value	Decision	Comment
QED	0.755	🟢	■ A measure of drug-likeness based on the concept of desirability;<br>■ Attractive: > 0.67;<br>■ unattractive: 0.49~0.67;<br>■ too complex: < 0.34
GASA	0.0	🟢	■ ES: Easy to synthesize; HS: Hard to synthesize; ■ The output value represents the probability of being difficult to synthesize, ranging from 0 to 1.
Synth	2.0	🟢	■ Synthetic accessibility score is designed to estimate ease of synthesis of drug-like molecules.<br>■ SAscore ≥ 6, difficult to synthesize; SAscore <6, easy to synthesize
Fsp3	0.333	🔴	■ The number of sp3 hybridized carbons / total carbon count, correlating with melting point and solubility.<br>■ Fsp^3 ≥0.42 is considered a suitable value.
MCE-18	30.0	🔴	■ MCE-18 stands for medicinal chemistry evolution.<br>■ MCE-18≥45 is considered a suitable value.
NPscore	-1.043	-	■ Natural product-likeness score.<br>■ This score is typically in the range from -5 to 5.<br>■ The higher the score is, the higher the probability is that the molecule is a NP.
Lipinski Rule	0.0	🟢	■ MW ≤ 500; logP ≤ 5; Hacc ≤ 10; Hdon ≤ 5<br>■ If two properties are out of range, a poor absorption or permeability is possible, one is acceptable.
Pfizer Rule	1.0	🔴	■ logP > 3; TPSA < 75<br>■ Compounds with a high log P (>3) and low TPSA (<75) are likely to be toxic.
GSK Rule	1.0	🔴	■ MW ≤ 400; logP ≤ 4<br>■ Compounds satisfying the GSK rule may have a more favorable ADMET profile
Golden Triangle	0.0	🟢	■ 200 ≤ MW ≤ 500; -2 ≤ logD ≤ 5<br>■ Compounds satisfying the Golden Triangle rule may have a more favorable ADMET profile.
PAINS	0 alerts	-	frequent hitters, Alpha-screen artifacts and reactive compound 480 substructures (J Med Chem 201053:2719-40)
ALARM NMR	2 alerts	-	Thiol reactive compounds.
BMS	0 alerts	-	undesirable, reactive compounds 176 substructures (J Chem Inf Model 200646:1060-8)
Chelator Rule	0 alerts	-	Chelating compounds.
Colloidal aggregators	0.366	-	■ Category 0: non-colloidal aggregators;<br>■ Category 1: colloidal aggregators.<br>■ The output value is the probability of being colloidal aggregators, within the range of 0 to 1.

FLuc inhibitors	0.002	🟢	■ Category 0: non-fLuc inhibitors;<br>■ Category 1: fLuc inhibitors.<br>■ The output value is the probability of being fLuc inhibitors, within the range of 0 to 1.
Blue fluorescence	0.004	🟢	■ Category 0: non-blue fluorescence;<br>■ Category 1: blue fluorescence.<br>■ The output value is the probability of being blue fluorescence, within the range of 0 to 1.
Green fluorescence	0.629	🔴	■ Category 0: non-green fluorescence;<br>■ Category 1: green fluorescence.<br>■ The output value is the probability of being green fluorescence, within the range of 0 to 1.
Reactive compounds	0.011	🟢	■ Category 0: non-reactive compound;<br>■ Category 1: reactive compound.<br>■ The output value is the probability of being reactive compound, within the range of 0 to 1.
Promiscuous compounds	0.165	🟢	■ Category 0: non-promiscuous compound;<br>■ Category 1: promiscuous compound.<br>■ The output value is the probability of being promiscuous compound, within the range of 0 to 1.

3. Absorption

Property	Value	Decision	Comment
Caco-2 Permeability	-4.788	🟢	Optimal: higher than -5.15 Log unit
MDCK Permeability	-4.613	🔴	■ low permeability: < 2 × 10-6 cm/s<br>■ medium permeability: 2-20 × 10^{-6} cm/s<br>■ high passive permeability: > 20 × 10^{-6} cm/s
PAMPA	0.0	🟢	■ The experimental data for Peff was logarithmically transformed (logPeff).<br>■ Molecules with log Peff values below 2.0 were classified as low-permeability (Category 0), while those with log Peff values exceeding 2.5 were classified as high-permeability (Category 1).
Pgp-inhibitor	0.972	🔴	■ Category 1: Inhibitor;<br>■ Category 0: Non-inhibitor;<br>■ The output value is the probability of being Pgp-inhibitor
Pgp-substrate	0.002	🟢	■ Category 1: substrate;<br>■ Category 0: Non-substrate;<br>■ The output value is the probability of being Pgp-substrate
HIA	0.0	🟢	■ Human Intestinal Absorption<br>■ Category 1: HIA+(HIA < 30%);<br>■ Category 0: HIA-(HIA >= 30%);<br>■ The output value is the probability of being HIA+

$F_{20\%}$	0.0	🟢	■ 20% Bioavailability<br>■ Category 1: F 20% + (bioavailability < 20%);<br>■ Category 0: F 20% - (bioavailability ≥ 20%);<br>■ The output value is the probability of being F 20% +
$F_{30\%}$	0.001	🟢	■ 30% Bioavailability<br>■ Category 1: F 30% + (bioavailability < 30%);<br>■ Category 0: F 30% - (bioavailability ≥ 30%);<br>■ The output value is the probability of being F 30% +
$F_{50\%}$	0.014	🟢	■ 50% Bioavailability<br>■ Category 1: F 50% + (bioavailability < 50%);<br>■ Category 0: F 50% - (bioavailability ≥ 50%);<br>■ The output value is the probability of being F 50% +

4. Distribution

Property	Value	Decision	Comment
PPB	94.476	🔴	■ Plasma Protein Binding<br>Optimal: < 90%.<br>■ Drugs with high protein-bound may have a low therapeutic index.
VDss	0.124	🟢	■ Volume Distribution<br>■ Optimal: 0.04-20L/kg
BBB	0.033	🟢	■ Blood-Brain Barrier Penetration<br>■ Category 1: BBB+; Category 0: BBB-;<br>■ The output value is the probability of being BBB+
Fu	4.785	🔴	■ The fraction unbound in plasms<br>■ Low: <5%; Middle: 5~20%; High: > 20%
OATP1B1 inhibitor	0.959	🔴	
OATP1B3 inhibitor	0.854	🔴	■ Category 0: Non-inhibitor; Category 1: inhibitor.<br>■ The output value is the probability of being inhibitor, within the range of 0 to 1.
BCRP inhibitor	0.622	🟠	■ Category 0: Non-inhibitor; Category 1: inhibitor.<br>■ The output value is the probability of being inhibitor, within the range of 0 to 1.
MRP1 inhibitor	0.945	🔴	■ Category 0: Non-inhibitor; Category 1: inhibitor.<br>■ The output value is the probability of being inhibitor, within the range of 0 to 1.

5. Metabolism

Property	Value	Decision	Comment
CYP1A2 inhibitor	0.989	🔴	■ Category 1: Inhibitor; Category 0: Non-inhibitor;<br>■ The output value is the probability of being inhibitor.

CYP1A2 substrate	1.0	🔴	■ Category 1: Substrate; Category 0: Non-substrate;<br>■ The output value is the probability of being substrate.
CYP2C19 inhibitor	0.999	🔴	■ Category 1: Inhibitor; Category 0: Non-inhibitor;<br>■ The output value is the probability of being inhibitor.
CYP2C19 substrate	0.213	🟢	■ Category 1: Substrate; Category 0: Non-substrate;<br>■ The output value is the probability of being substrate.
CYP2C9 inhibitor	0.983	🔴	■ Category 1: Inhibitor; Category 0: Non-inhibitor;<br>■ The output value is the probability of being inhibitor.
CYP2C9 substrate	0.997	🔴	■ Category 1: Substrate; Category 0: Non-substrate;<br>■ The output value is the probability of being substrate.
CYP2D6 inhibitor	0.711	🔴	■ Category 1: Inhibitor; Category 0: Non-inhibitor;<br>■ The output value is the probability of being inhibitor.
CYP2D6 substrate	0.012	🟢	■ Category 1: Substrate; Category 0: Non-substrate;<br>■ The output value is the probability of being substrate.
CYP3A4 inhibitor	0.007	🟢	■ Category 1: Inhibitor; Category 0: Non-inhibitor;<br>■ The output value is the probability of being inhibitor.
CYP3A4 substrate	0.997	🔴	■ Category 1: Substrate; Category 0: Non-substrate;<br>■ The output value is the probability of being substrate.
CYP2B6 inhibitor	0.017	🟢	■ Category 1: Inhibitor; Category 0: Non-inhibitor;<br>■ The output value is the probability of being inhibitor.
CYP2B6 substrate	0.001	🟢	■ Category 1: Substrate; Category 0: Non-substrate;<br>■ The output value is the probability of being substrate.
CYP2C8 inhibitor	1.0	🔴	■ Category 1: Inhibitor; Category 0: Non-inhibitor;<br>■ The output value is the probability of being inhibitor.
HLM Stability	0.847	🔴	■ human liver microsomal (HLM) stability<br>■ Category 0: stable+ (HLM > 30 min); Category 1: unstable- (HLM ≤ 30 min). The output value is the probability of human liver microsomal instability, where a value closer to 1 indicates a higher likelihood of instability. The range is between 0 and 1.

6. Excretion

CL_{plasma}	8.19	●	■ The unit of predicted CLplasma penetration is ml/min/kg. >15 ml/min/kg: high clearance; 5-15 ml/min/kg: moderate clearance; < 5 ml/min/kg: low clearance.
$T_{1/2}$	0.782	●	■ The unit of predicted T1/2 is hours. ■ ultra-short half-life drugs: 1/2 < 1 hour; short half-life drugs: T1/2 between 1-4 hours; intermediate short half-life drugs: T1/2 between 4-8 hours; long half-life drugs: T1/2 > 8 hours.

7. Toxicity

Property	Value	Decision	Comment
hERG Blockers	0.844	●	■ Molecules with IC50 ≤10μM or ≥50% inhibition at 10 μM were classified as hERG+ (Category 1), ■ while molecules with IC50 >10μM or < 50% inhibition at 10μM were classified as hERG (Category 0). ■ The output value is the probability of being hERG+, within the range of 0 to 1.
hERG Blockers (10um)	0.797	●	■ Molecules with IC50 ≤10 μM are classified as hERG+ (Category 1), ■ and molecules with IC50 > 10μM are classified as hERG- (Category 0). ■ The output value is the probability of being hERG+, within the range of 0 to 1.
DILI	0.879	●	■ Drug Induced Liver Injury. ■ Category 1: drugs with a high risk of DILI; ■ Category 0: drugs with no risk of DILI. ■ The output value is the probability of being toxic.
AMES Mutagenicity	0.086	●	■ AMES Toxicity ■ Category 1: Ames positive(+); ■ Category 0: Ames negative(-); ■ The output value is the probability of being toxic.
Rat Oral Acute Toxicity	0.835	●	■ Rat Oral Acute Toxicity. ■ Category 0: low-toxicity, > 500 mg/kg; ■ Category 1: high-toxicity; < 500 mg/kg. ■ The output value is the probability of being toxic, within the range of 0 to 1.
FDAMDD	0.911	●	■ FDA Maximum (Recommended) Daily Dose. ■ Category 1: FDAMDD (+); ■ Category 0: FDAMDD (-); The output value is the probability of being positive.
Skin Sensitization	0.245	●	■ Category 1: Sensitizer; ■ Category 0: Non-sensitizer. ■ The output value is the probability of being toxic, within the range of 0 to 1.
Carcinogenicity	0.315	●	■ Category 1: carcinogens; ■ Category 0: non-carcinogens; ■ The output value is the probability of being toxic.

Eye Corrosion	0.0	●	■ Eye Corrosion<br>■ Category 1: corrosives;<br>Category 0: noncorrosives;<br>■ The output value is the probability of being corrosives.
Eye Irritation	0.238	●	■ Eye Irritation<br>■ Category 1: irritants;<br>Category 0: nonirritants;<br>■ The output value is the probability of being irritants.
Respiratory	0.985	●	■ Category 1: respiratory toxicants; ■ Category 0: non-respiratory toxicants.<br>■ The output value is the probability of being toxic, within the range of 0 to 1.
Human Hepatotoxicity	0.868	●	■ Human Hepatotoxicity<br>■ Category 1: H-HT positive(+);<br>■ Category 0: H-HT negative(-);<br>■ The output value is the probability of being toxic.
Drug-induced Nephrotoxicity	0.997	●	■ Category 0: non-nephrotoxic (-);<br>■ Category 1: nephrotoxic (+).<br>■ The output value is the probability of being nephrotoxic (+), within the range of 0 to 1.
Ototoxicity	0.902	●	■ Category 0: non-ototoxicity (-);<br>■ Category 1: ototoxicity (+).<br>■ The output value is the probability of being ototoxicity (+), within the range of 0 to 1.
Hematotoxicity	0.453	●	■ Category 0: non-hematotoxicity (-);<br>■ Category 1: hematotoxicity (+).<br>■ The output value is the probability of being hematotoxicity (+), within the range of 0 to 1.
Genotoxicity	0.999	●	■ Category 0: non-Genotoxicity (-);<br>■ Category 1: Genotoxicity (+).<br>■ The output value is the probability of being ototoxicity (+), within the range of 0 to 1.
RPMI-8226 Immunitoxicity	0.083	●	■ Category 0: non-cytotoxicity (-);<br>■ Category 1: cytotoxicity (+).<br>■ The output value is the probability of being ototoxicity (+), within the range of 0 to 1.
A549 Cytotoxicity	0.88	●	■ Category 0: non-cytotoxicity (-);<br>■ Category 1: cytotoxicity (+).<br>■ The output value is the probability of being ototoxicity (+), within the range of 0 to 1.
Hek293 Cytotoxicity	0.876	●	■ Category 0: non-cytotoxicity (-);<br>■ Category 1: cytotoxicity (+).<br>■ The output value is the probability of being ototoxicity (+), within the range of 0 to 1.
Drug-induced Neurotoxicity	0.941	●	■ Category 0: non-neurotoxic (-);<br>■ Category 1: neurotoxic (+).<br>■ The output value is the probability of being neurotoxic (+), within the range of 0 to 1.

8. Environmental toxicity

Property	Value	Comment
Bioconcentration Factors	2.551	■ Bioconcentration factors are used for considering secondary poisoning potential and assessing risks to human health via the food chain.<br>■ The unit is −log10[(mg/L)/(1000*MW)]
IGC_{50}	3.807	■ Tetrahymena pyriformis 50 percent growth inhibition concentration.<br>■ The unit is −log10[(mg/L)/(1000*MW)]
$LC_{50}FM$	5.14	■ 96-hour fathead minnow 50 percent lethal concentration.<br>■ The unit is −log10[(mg/L)/(1000*MW)]
$LC_{50}DM$	5.968	■ 48-hour daphnia magna 50 percent lethal concentration.<br>■ The unit is −log10[(mg/L)/(1000*MW)]

9. Tox21 pathway

Property	Value	Decision	Comment
NR-AhR	0.557	●	■ Aryl hydrocarbon receptor<br>■ Category 1: actives ;<br>■ Category 0: inactives;<br>■ The output value is the probability of being active.
NR-AR	0.02	●	■ Androgen receptor<br>■ Category 1: actives ;<br>■ Category 0: inactives;<br>■ The output value is the probability of being active.
NR-AR-LBD	0.0	●	■ Androgen receptor ligand-binding domain<br>■ Category 1: actives ;<br>■ Category 0: inactives;<br>■ The output value is the probability of being active.
NR-Aromatase	0.766	●	■ Category 1: actives ;<br>■ Category 0: inactives;<br>■ The output value is the probability of being active.
NR-ER	0.312	●	■ Estrogen receptor<br>■ Category 1: actives ;<br>■ Category 0: inactives;<br>■ The output value is the probability of being active.
NR-ER-LBD	0.005	●	■ Estrogen receptor ligand-binding domain<br>■ Category 1: actives ;<br>■ Category 0: inactives;<br>■ The output value is the probability of being active.
NR-PPAR-gamma	0.004	●	■ Peroxisome proliferator-activated receptor gamma<br>■ Category 1: actives ;<br>■ Category 0: inactives;<br>■ The output value is the probability of being active.

SR-ATAD5	0.027	●	■ ATPase family AAA domain-containing protein 5<br>■ Category 1: actives ;<br>■ Category 0: inactives;<br>■ The output value is the probability of being active.
SR-HSE	0.001	●	■ Heat shock factor response element<br>■ Category 1: actives ;<br>■ Category 0: inactives;<br>■ The output value is the probability of being active.
SR-MMP	0.244	●	■ Mitochondrial membrane potential<br>■ Category 1: actives ;<br>■ Category 0: inactives;<br>■ The output value is the probability of being active.
SR-p53	0.058	●	■ p53, a tumor suppressor protein<br>■ Category 1: actives ;<br>■ Category 0: inactives;<br>■ The output value is the probability of being active.

10. Toxicophore Rules

Property	Value	Comment
Acute Toxicity Rule	0	■ 20 substructures;<br>■ acute toxicity during oral administration
Genotoxic Carcinogenicity Rule	1 alerts	■ 117 substructures;<br>■ carcinogenicity or mutagenicity
NonGenotoxic Carcinogenicity Rule	0	■ 23 substructures;<br>■ carcinogenicity through nongenotoxic mechanisms
Skin Sensitization Rule	2 alerts	■ 155 substructures;<br>■ skin irritation
Aquatic Toxicity Rule	4 alerts	■ 99 substructures;<br>■ toxicity to liquid(water)
NonBiodegradable Rule	2 alerts	■ 19 substructures;<br>■ non-biodegradable
SureChEMBL Rule	0	■ 164 substructures;<br>■ MedChem unfriendly status
FAF-Drugs4 Rule	0	154 toxic substructures from FAF-Drug4

SR-ARE	0.543	●	■ Antioxidant response element<br>■ Category 1: actives ;<br>■ Category 0: inactives;<br>■ The output value is the probability of being active.
